# *Clostridium difficile* infection among hospitalized HIV-infected individuals: epidemiology and risk factors: results from a case-control study (2002-2013)

**DOI:** 10.1186/s12879-015-0932-x

**Published:** 2015-04-22

**Authors:** Stefano Di Bella, Alexander W Friedrich, Esther García-Almodóvar, Maria Serena Gallone, Fabrizio Taglietti, Simone Topino, Vincenzo Galati, Emma Johnson, Silvia D’Arezzo, Nicola Petrosillo

**Affiliations:** National Institute for Infectious Diseases “L. Spallanzani”, Via Portuense 292, 00149 Rome, Italy; Department of Medical Microbiology and Infection Control, University Medical Centre, Groeningen, The Netherlands; Department of Internal Medicine, Manacor Hospital, Palma of Majorca, Spain; Department of Biomedical Science and Human Oncology, Aldo Moro University of Bari, Bari, Italy; University of Sheffield, Sheffield, UK

**Keywords:** *Clostridium difficile*, HIV, AIDS, Case-control, Risk factors, Albumin, Gammaglobulins

## Abstract

**Background:**

HIV infection is a risk factor for *Clostridium difficile* infection (CDI) yet the immune deficiency predisposing to CDI is not well understood, despite an increasing incidence of CDI among such individuals. We aimed to estimate the incidence and to evaluate the risk factors of CDI among an HIV cohort in Italy.

**Methods:**

We conducted a retrospective case-control (1:2) study. Clinical records of HIV inpatients admitted to the National Institute for Infectious Disease “L. Spallanzani”, Rome, were reviewed (2002-2013). Cases: HIV inpatients with HO-HCFA CDI, and controls: HIV inpatients without CDI, were matched by gender and age. Logistic regression was used to identify risk factors associated with CDI.

**Results:**

We found 79 CDI episodes (5.1 per 1000 HIV hospital admissions, 3.4 per 10000 HIV patient-days). The mean age of cases was 46 years. At univariate analysis factors associated with CDI included: antimycobacterial drug exposure, treatment for Pneumocystis pneumonia, acid suppressant exposure, previous hospitalization, antibiotic exposure, low CD4 cell count, high Charlson score, low creatinine, low albumin and low gammaglobulin level. Using multivariate analysis, lower gammaglobulin level and low serum albumin at admission were independently associated with CDI among HIV-infected patients.

**Conclusions:**

Low gammaglobulin and low albumin levels at admission are associated with an increased risk of developing CDI. A deficiency in humoral immunity appears to play a major role in the development of CDI. The potential protective role of albumin warrants further investigation.

## Background

The incidence of *Clostridium difficile* infection (CDI) is increasing worldwide in both the general population and immunocompromised individuals [1-4].

Several factors have been associated with the risk of CDI development, including: older age, antibiotic exposure, acid suppressant exposure, inflammatory bowel disease and immunosuppression [5-9]. Immune system disorders are commonly documented as risk factors for CDI and despite the advent of HAART, HIV seropositive individuals still represent a large immunosuppressed population. HIV infection has been found to be a risk factor for CDI [5]. The role of cellular immunity in the development of opportunistic infections is increasingly understood, yet the immune deficiency predisposing HIV-infected individuals to CDI has not been adequately studied. There are no studies of CDI incidence in HIV cohorts in Europe from the later HAART era (i.e. post 2002); a time period that also represents a considerable change in CDI epidemiology, including the spread of hypervirulent strains and the emergence of increasing resistance rates to antimicrobials, in developed countries [10-12].

Our aim was to estimate the incidence of CDI among HIV hospitalized patients, and to evaluate the associated risk factors.

## Methods

We collected data from 2002 to 2013 on CDI among HIV-infected inpatients in our hospital, National Institute for Infectious Diseases “L. Spallanzani”, that is a a referral center for HIV infected individuals in our region of approximately 5.5 million inhabitants. CDI cumulative incidence was expressed as CDI episodes per 1000 hospital admissions of HIV-infected patients. CDI incidence rate was expressed as the number of CDI episodes per 10000 patient-days among HIV-infected inpatients. Also data on CDI among non HIV-infected inpatients were collected in the same period, in order to compare the CDI trend in both the groups, HIV-infected and not infected. In this case CDI cumulative incidence was expressed as CDI episodes per 1000 hospital admissions of non-HIV infected patients and CDI incidence rate was expressed as the number of CDI episodes per 10000 patient-days among non HIV-infected inpatients.

Moreover, we conducted a retrospective case-control (1:2) study on adult HIV-infected patients. Cases were defined as HIV inpatients with CDI; controls were HIV inpatients without CDI that were hospitalized for at least 48 hours. Controls were patients without diarrhea or with diarrhea but with negative toxin test for *C. difficile*. Controls were randomly selected and matched for gender, age (±5 years) and year of admission with cases.

Only healthcare facility (HCF)-onset, HCF-associated (HO-HCFA) CDI were included in the study. HO-HCFA CDI is defined as an episode of CDI occurring between 48 hours after admission and hospital discharge [13].

A CDI episode was considered as a positive *C. difficile* toxin assay in a stool sample from a patient with diarrhea. Diarrhea was defined as ≥ 3 unformed stools in a 24-hour period. The presence of toxin A and B was tested through enzyme immunoassays (EIA) for A/B toxins (*C. difficile* Tox A/B, TechLab, Blacksburg, VA). CDI relapses were excluded from the analysis.

Demographic data (gender, age), fever (i.e. temperature > 38.3°C) and biochemical parameters (white blood cell count, serum gammaglobulin level, serum albumin level, serum creatinine level, CD4 cell count) measured at admission were recorded. Gammaglobulin levels were calculated through seroprotein electrophoresis from total proteins. Normal range for serum albumin was 3.5-5.5 g/dl and for serum gammaglobulin 800-1600 mg/dl according to our laboratory. In addition the following characteristics were compared between cases and controls: Charlson’s score index, length of hospital stay, antiretroviral treatment, antimycobacterial drug exposure, prophylaxis with trimethoprim-sulfamethoxazole (TMP-SMX) and/or azithromycin, chronic hepatitis C virus (HCV) infection, treatment for Pneumocystis pneumonia, antineoplastic chemotherapy, acid suppressants, prior hospitalization (in the previous 8 weeks), prior antibiotic exposure (in the previous 8 weeks), antibiotic exposure during hospitalization and methadone treatment. The recorded data were available for all cases and controls.

The study involved the analysis of existing clinical and laboratory data that were anonymised before being included in the study database. The study was approved by the Ethics Committee of the National Institute for Infectious Diseases “L. Spallanzani”.

Continuous variables are expressed as mean ± SD and categorical variables as percentages.

Univariate analysis was performed for each recorded variable, with the exception of mortality and length of stay since such variables could be both a risk factor and/or a consequence of CDI. Odds ratio (OR), with 95% confidence interval, was calculated for qualitative variables; quantitative variables with normal distribution were compared using the Student’s t test.

Multivariate regression analysis included all statistically significant variables in univariate analysis and all clinically relevant variables, whether statistically significant or not [14]. The final model included the following variables: antimycobacterial drugs exposure, treatment for Pneumocystis pneumonia, acid suppressant use, previous hospitalization, prior antibiotic exposure, antibiotic exposure during hospital stay, CD4 cell count on admission, Charlson score, serum creatinine on admission, serum albumin on admission and gammaglobulin level on admission.

The Hosmer and Lemeshow test was applied to estimate the goodness of fit for the model.

The statistical significance was set at P < 0,05.

The analysis was performed using STATA 11 MP (StataCorp LP, College Station, Texas).

## Results

**Figure 1:**
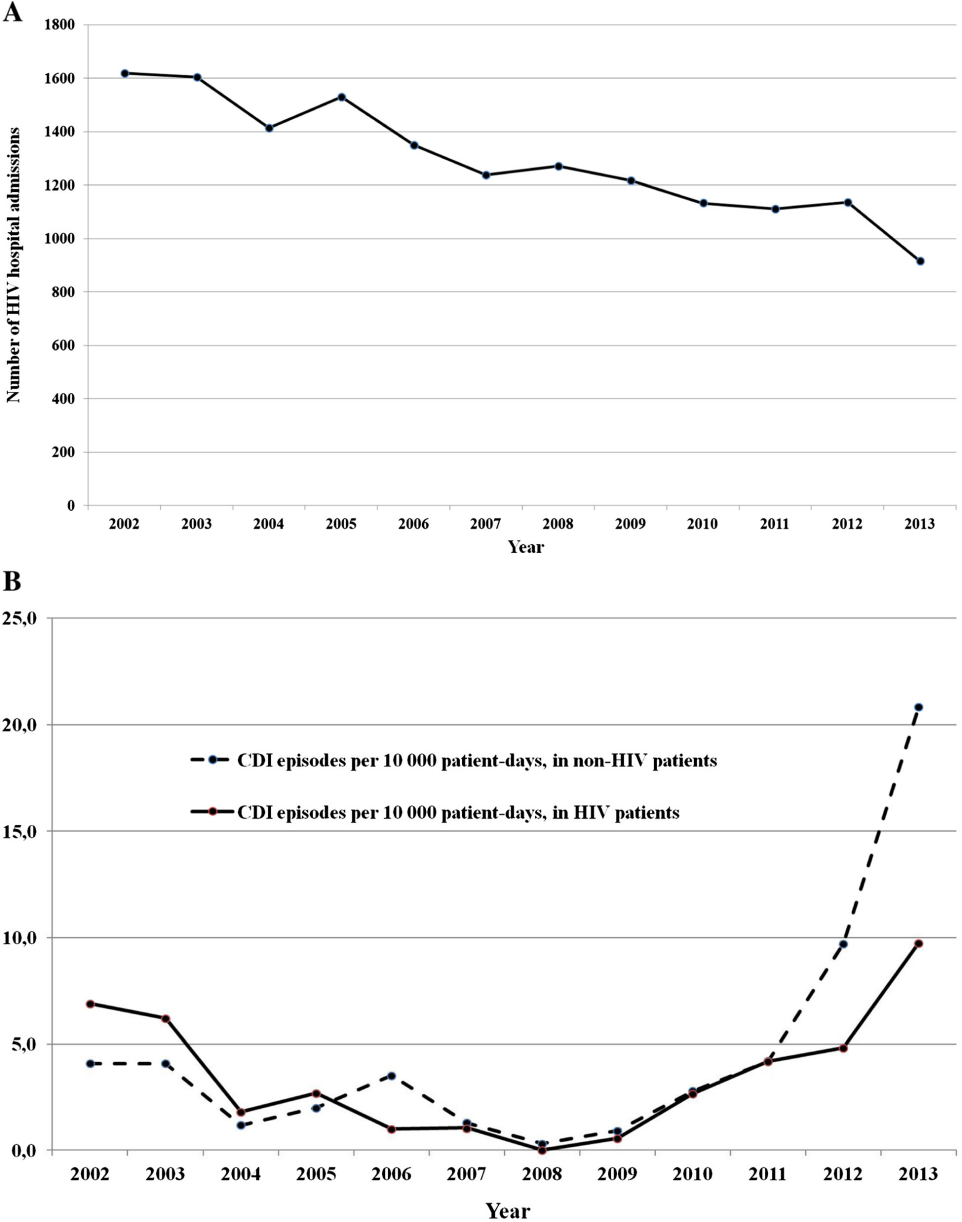
HIV admissions and CDI episodes: trends over time. **A** – HIV admissions per year; **B** – CDI episodes per year in HIV and non-HIV patients.

From January 2002 to December 2013, 15 537 HIV-infected patients were admitted to our Institute. The distribution of HIV admission during this time is shown in Figure 1A.

Seventy-nine cases of HO-HCFA CDI were identified among HIV-infected inpatients during the study period. Cumulative incidence was 5.1 per 1000 HIV hospital admissions, with an incidence rate of 3.4 CDI cases per 10,000 HIV patient-days. Figure 1B compares the trend of CDI incidence among HIV and non-HIV inpatients through the study period.

Among CDI cases, 56 were male (71%), 23 were female (29%) and the mean age was 46 years. Forty-three percent of CDI cases had fever at diagnosis of CDI and the mean white cell count at diagnosis was 7061 per cubic millimeter (SD ±5167).

Regarding antibiotic exposure, carbapenem, fluoroquinolone and rifamycin exposures were significantly more prevalent among cases compared to controls (Table 1).

**Table 1:** **Antibiotics taken before and during hospital stay, cases and controls**

**Antibiotics before hospital stay**	**Cases (%) (n = 79)**	**Controls (%) (N = 158)**	**OR (95% CI)**	**p**
**Fluoroquinolones**	11 (14)	7 (4)	3.5 (1.2 – 11.0)	0.009*
**3** ^**rd**^ **/4** ^**th**^ **gen. Cephalosporins**	6 (7)	12 (8)	1 (0.3 – 3.0)	1
**Carbapenems**	1 (1)	2 (1)	1 (0.0 – 19.5)	1
**Penicillins**	4 (5)	12 (8)	0.6 (0.1 – 2.24)	0.464
**Rifamycins**	8 (10)	6 (4)	2.9 (0.8 – 10.3)	0.05*
**Clindamycin**	0 (0)	0 (0)	\	\
**Antibiotics during hospital stay**				
**Fluoroquinolones**	28 (35)	29 (18)	2.4 (1.3 – 4.7)	0.004*
**3** ^**rd**^ **/4** ^**th**^ **gen. Cephalosporins**	25 (32)	53 (34)	0.9 (0.5 – 1.7)	0.77
**Carbapenems**	18 (23)	9 (6)	4.9 (1.9 – 13.0)	0.0001*
**Penicillins**	15 (19)	27 (17)	1.1 (0.5 – 2.4)	0.718
**Rifamycins**	11 (14)	8 (5)	3.0 (1.1 – 9.1)	0.18
**Clindamycin**	3 (4)	1 (1)	6.2 (0.5 – 327.3)	0.075

*p ≤ 0.05.

Regimens used to treat CDI were metronidazole alone for 42 patients (53%), oral vancomycin alone for 11 patients (14%). Fourteen patients received both vancomycin and metronidazole (18%) and 12 patients (15% of cases) received no CDI treatment during the hospital stay because of death (1 patient) or voluntary discharge (11 patients).

The mean duration of diarrhea among CDI cases was 13 days (SD ±12).

**Figure 2:**
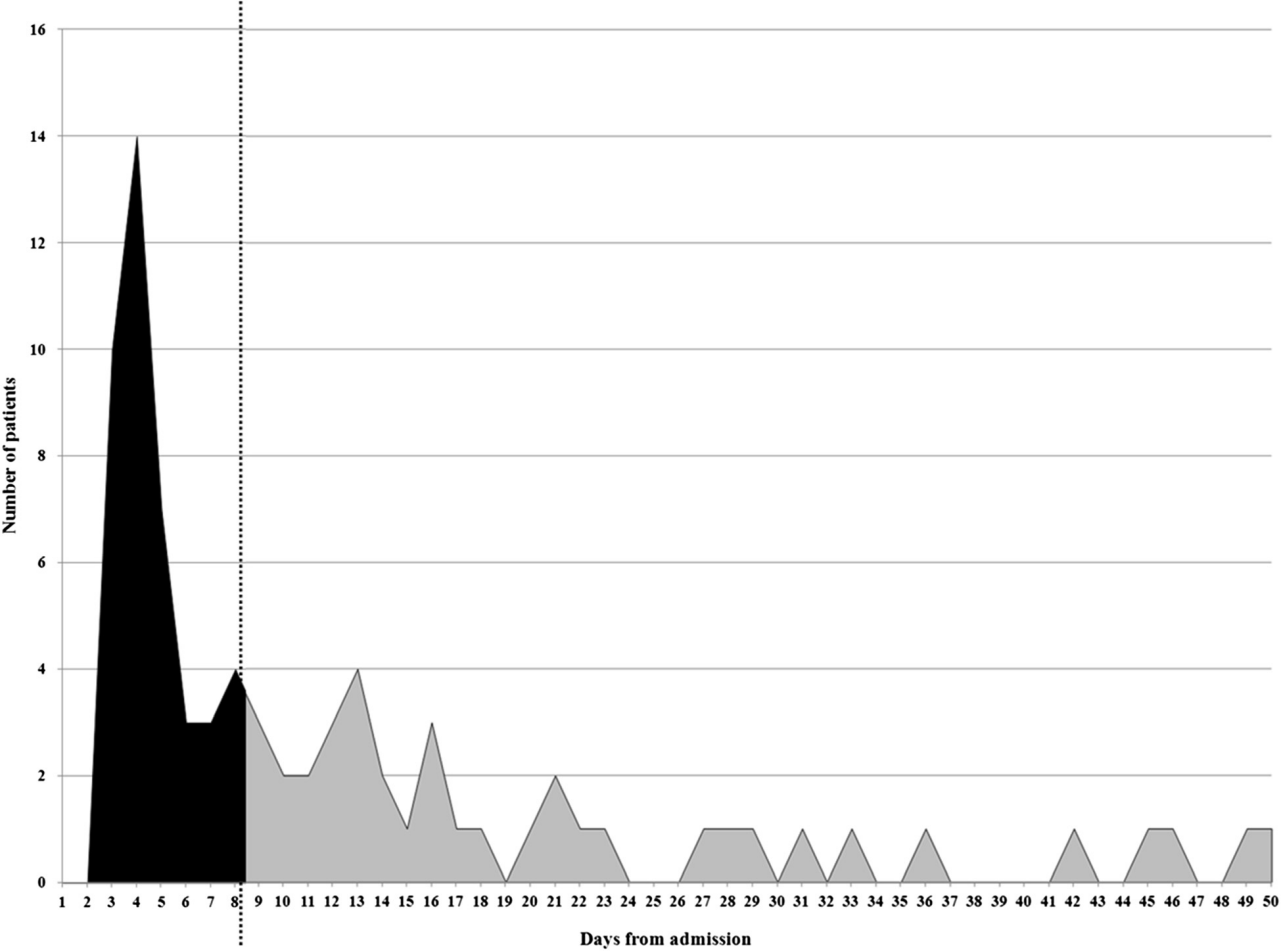
Time to onset of diarrhea among hospitalized HIV patients with CDI.

CDI occurred, on average, 12.7 days after admission (range 3-50 days, SD ±11.8), with a median of 8 days after the admission (IQR 4-16). The time to onset of diarrhea during the hospital stay (from 48 hours after the admission to the discharge) is illustrated in Figure 2. In just over half of the patients (51.8%) (Figure 2, dashed line) CDI occurred during the first 8 days after hospital admission.

The risk factors analyzed for the 79 CDI cases and 158 matched control subjects are shown in Table 2. In univariate analysis the following variables were significantly associated with the development of CDI: antimycobacterial drug exposure, treatment for Pneumocystis pneumonia, acid suppressant exposure, previous hospitalization, prior antibiotic exposure, antibiotic exposure during hospital stay, low CD4 cell count, high Charlson score, low serum creatinine level, low serum albumin level and low gammaglobulin level.

**Table 2:** **Demographic and clinical characteristics of incident CDI, cases and matched controls**

**Characteristic**	**Categories**	**Cases (N = 79)**	**Controls (N = 158)**	**Total (N = 237)**	**Unadjusted comparison**	**P value**
**Age, mean (years)**		45,9 ± 9,3	45,4 ± 8,8	45,5 ± 8,9	MD – 0,6 (±1,2)	0,64
**Gender, N (%)**	M	56 (70,1)	116 (73,4)	172 (72,6)		
	F	23 (29,1)	42 (26,6)	65 (27,4)		
**Hospitalization (days)^**		33,5 ± 26,6	14,2 ± 12,2	20,6 ± 20,3		
**Antiretroviral Treatment**		55 (69,6)	96 (60,8)	151 (63,7)	OR 1,5 (0,8 – 2,8)	0,20
**Chronic HCV**		39 (49,4)	67 (42,4)	106 (44,7)	OR 1,3 (0,7 – 2,4)	0,31
**Acid suppressants**		36 (45,6)	51 (32,3)	87 (36,7)	OR 1,8 (1,0 – 3,2)	0,04*
**Antineoplastic chemotherapy**		9 (11,4)	11 (7,0)	20 (8,4)	OR 1,7 (0,6 – 4,8)	0,25
**Previous hospitalization** ^**§**^		45 (57,0)	55 (34,8)	100 (42,2)	OR 2,5 (1,4 – 4,5)	0,01*
**Antibiotic exposure**		71	123	194	OR 2,5 (1,1 – 6,6)	0,02*
**Death during hospitalization**		11 (13,9)	10 (6,3)	21 (8,9)		
**Methadone therapy**		17 (21,5)	37 (23,4)	54 (22,8)	OR 0,9 (0,4 – 1,8)	0,74
**CD4 on admission^**		188 ± 237	262 ± 259	238 ± 254	MD 74,3 (±34,8)	0,03*
**Charlson score^**		6,8 ± 3,4	5,7 ± 3,7	6,1 ± 3,6	MD -1,1 (±0,5)	0,027*
**HIV RNA, median**		20.610	2.214	3.601	MD -15.935 (±33.975)	0,640
**Serum Creatinine on admission (mg/dl)^**		1,3 ± 1,8	0,9 ± 0,7	1,1 ± 1,2	MD 1,1 (±0,1)	0,036*
**Serum Albumin on admission (g/dl)^**		3,0 ± 0,7	3,4 ± 0,7	3,2 ± 0,7	MD 0,4 (±0,1)	<0,001*
**Gammaglobulins on admission (g/dl)^**		1,6 ± 0,7	1,9 ± 1,0	1,8 ± 0,9	MD 0,2 (±0,1)	0,05*

Extreme values (outliers: 2 upper and 2 lower) were excluded; there are no missing data; **^**: mean; ^§^: 8 weeks before; *: P value ≤ 0,05; Antibiotic exposure was evaluated until the development of diarrhea among cases and until the discharge for controls; MD: mean difference and standard deviation; OR: Odds ratios and 95% confidence intervals; P: prophylaxis; PCP: Pneumocystis pneumonia; TMP-SMX: trimethoprim-sulfamethoxazol.

Univariate analysis for factors associated with CDI in HIV-infected hospitalized individuals

On multivariable analysis (Table 3) low gammaglobulin level and low serum albumin on admission were independent factors associated with the development of CDI among HIV infected individuals during hospitalization.

**Table 3:** **Independent risk factors for CDI among HIV-infected inpatients as defined by multivariate logistic regression**

**Variable**	**OR**	**(95% CI)**	**P**
**Acid suppressants**	1.23	0.67-2.26	0.49
**Previous hospitalization (<8 weeks)**	1.69	0.92-3.11	0.09
**Antibiotic exposure**	2.02	0.83-4.91	0.12
**CD4 cell count on admission**	0.99	0.99-1.00	0.25
**Charlson score**	1.02	0.94-1.12	0.53
**Serum Creatinine on admission (mg/dl)**	1.09	0.86-1.39	0.45
**Serum Albumin on admission (g/dl)**	0.61	0.39-0.96	0.03*
**Gammaglobulins on admission (g/dl)**	0.68	0.48-0.96	0.03*

Antibiotic exposure was evaluated until the development of diarrhea among cases and until hospital discharge for controls. CI: confidence intervals; OR: odds ratio; * p < 0.05.

Eleven patients (13.9%) among cases and 10 patients (6.3%) among controls died during the hospital stay (p = 0.05). In 3 patients, in-hospital death was attributed to CDI.

## Discussion

HIV infection is an established risk factor for CDI and the introduction of HAART has not effectively reduced the risk of *C. difficile* among hospitalized HIV-infected patients [15,16]. Although the incidence of HO-HCFA that we found is lower (approximately 10 fold) as compared to pre-HAART studies [17], in this study, we found an increasing incidence in HO-HCFA CDI cases among HIV infected patients admitted to our Institute from 2008 to 2013, yet the number of admissions for HIV related infections has been progressively decreasing. Moreover we found that CD4 count was not associated with CDI occurrence, whereas a lower level of gammaglobulins was independently associated. Hypogammaglobulinemia is a known predisposing factor for CDI in the non-HIV population but an association has not been evaluated in the HIV population [18-20].

However, in a study published in 1997, Barbut *et al*. found that CD4 count was significantly associated with CDI among HIV-infected patients, but gammaglobulin levels were not evaluated among risk factors [21]. Similarly in another recent study conducted in United States evaluating the risk factors associated with CDI development among HIV infected individuals, gammaglobulin levels were not considered among variables to be assessed as risk factors [4].

Host immunity, especially humoral responses to *C. difficile* toxins, is now believed to be a major determinant of the consequences of *C. difficile* acquisition [22-24]. Indeed, in solid organ transplanted patients severe hypogammaglobulinemia has been demonstrated to be a risk factor for developing CDI [19].

Variation in antibody response may be a principal explanation for the association of symptomatic *C. difficile* infections with advanced age and immunodeficiency-associated conditions, including HIV infection [25]. HIV infection deranges both cellular and humoral immunity and antigen-specific B cell memory responses are severely impaired among HIV-infected individuals [26,27]. The ability of B cells to release immunoglobulins in vitro has been shown to be dependent on functional CD4 cells [28,29]. In studies which have demonstrated an association between low CD4 cell count and CDI occurrence it is possible that the concurrently impaired humoral immunity plays a role. Indeed, in our multivariate analysis, when all potential risk factors, including both CD4 cell count and gammaglobulin levels, are taken into account, CD4 cell count *per se* is not significantly associated with development of CDI [4,21]. Therefore, we hypothesise that the measurement of CD4 cell count alone is serving as a proxy marker for impaired humoral immunity and that it is the latter which may be truly responsible for the predisposition of HIV infected patients to acquire *C. difficile* disease.

In the 1990s, *C. difficile* strains were often sensitive to rifamycins (rifampicin, rifabutin) [30], yet more recently there is growing evidence to show that several *C. difficile* strains, mainly ribotype 027, are resistant to rifamycins [31]. Therefore, the clinical use of such class of antibiotics could be a potential risk factor for CDI. In particular, given the spread of ribotype 027 in Italy [32] we think it is important to reassess the risk of CDI in patients receiving antimycobacterial treatment. In our study 19% of cases and 8% of controls were exposed to antimycobacterial treatment (p = 0.02). Although on multivariate analysis, “antimycobacterial drug exposure” as a risk factor for CDI did not reach statistical significance, we think that further studies should address this potential association.

Low serum albumin is a well known risk factor for CDI [33,34]. In our study albumin levels measured on admission were lower in cases compared to controls (3 g/dl vs 3.4 g/dl), with a significant p-value at multivariate analysis (p = 0.03). Since we assessed serum albumin levels on admission, low albumin is not a consequence of protein-losing enteropathy (i.e. low albumin is not secondary to CDI) and should be considered a possible predisposing factor for CDI development. Indeed, low albumin could represent a marker of poor conditional status (i.e. cachexia, malnutrition, wasting syndrome, cirrhosis, nephritic syndrome) [35]. Similar findings have been recently reported by Kumarappa *et al*. in non-HIV patients [36].

In this study, we observed a reduction in CDI incidence from 2002 to 2008 and a progressive increase from 2008 to 2013 that was similar to that observed among non-HIV patients hospitalized between 2008 and 2013 (see Figure 1). We speculate that the reduction observed in the first period reflects, in part, the observed decrease in HIV patient admissions and partially to a reduction of opportunistic infections [37] and therefore, antibiotic therapy. However, we hypothesize that the increasing incidence observed from 2008 to 2013, in light of the continued decrease in number of hospitalizations among HIV patients, could be related to the emergence of the *C. difficile* strain BI/NAP1/027. In support of this, the first case reports of *C. difficile* 027 in Italy were published in January 2010 [38] and in 2012 data from a small case series demonstrated that 59% of the analyzed strains were ribotype 027 [32].

Whilst this case:control study has yielded important insights into potential mechanisms for the increased propensity to CDI in HIV infected patients, our retrospective study has some limitations. We only considered one episode of CDI per patient thus recurrent disease was not evaluated. We did not evaluate community acquired CDI, but we must consider that different risk factors may be important outside the nosocomial environment. In our hospital, the number of diagnostic tests for CDI increased during the study period (in particular from 2006), therefore CDI episodes since this time could be potentially over-represented. In addition we only used the EIA toxins detection as diagnostic test for CDI; this test has a low sensitivity therefore it is possible that patients with true CDI but with false negative toxin EIA may have been inappropriately included as control. Moreover we did not perform culture for *C. difficile* and we could not ascertain if the CDI rise has been due to one/few clonal lineages of *C. difficile*. Finally, selection bias is a risk in case:control studies and we strived to minimize this by including all known CDI cases during the study period and random selection of controls.

## Conclusions

In our study, lower gammaglobulin and albumin levels are associated with the developing of CDI. It remains vital that the immune constitutents that play a role in susceptibility to CDI are further elucidated. Strategies to improve humoral immunity and albumin status in patients with immune dysfunction should be further explored in an effort to reduce the incidence of CDI.
